# Serum antibody response to BNT162b2 after natural SARS‐CoV‐2 infection

**DOI:** 10.1111/eci.13632

**Published:** 2021-08-01

**Authors:** Thomas Perkmann, Nicole Perkmann‐Nagele, Thomas Koller, Patrick Mucher, Astrid Radakovics, Michael Wolzt, Oswald F. Wagner, Christoph J. Binder, Helmuth Haslacher

**Affiliations:** ^1^ Department of Laboratory Medicine Medical University of Vienna Vienna Austria; ^2^ Department of Clinical Pharmacology Medical University of Vienna Vienna Austria

**Keywords:** antibody response, SARS‐CoV‐2, serology, seropositive, vaccination

## Abstract

**Background:**

There is preliminary evidence that individuals with previous SARS‐CoV‐2 infections exhibit a more pronounced antibody response. However, these assumptions have not yet been supported by data obtained through various CE‐marked tests. This study aimed to close this gap.

**Methods:**

Sixty‐nine seronegatives and 12 individuals post‐SARS‐CoV‐2 infection (tested by CE‐labelled Roche NC immunoassay or PCR‐confirmed assay) were included 21 ± 1 days after receiving the first dose of the Pfizer/BioNTech BNT162b2 vaccine. Antibody response to viral spike protein (S) was assessed by CE‐labelled Roche S and DiaSorin S1/S2 assays and by a surrogate virus neutralization test (sVNT).

**Results:**

After a single dose of BNT162b2, individuals after natural SARS‐CoV‐2 infection presented with markedly higher anti‐S levels than naïve individuals (Roche S: 9078.5 BAU/mL [5267.0‐24 298.5] vs 79.6 [24.7‐142.3]; and DiaSorin S1/S2: 1465.0 AU/mL [631.0‐5365.0] vs 63.7 [47.8‐87.5]) and showed all the maximum observed inhibition activity in the sVNT (98%), without overlaps between groups. There was a trend for higher responses in those with a more distant infection, although not statistically significant. The relative antibody increase after dose 2 was significantly higher among naïve individuals (25‐fold), but antibody levels remained below that of seropositives.

**Conclusions:**

Compared with naïve individuals, seropositives after natural SARS‐CoV‐2 infection presented with a substantially higher antibody response already after dose 1 of BNT162b2, as measured by two CE‐marked in vitro diagnostic tests and a sVNT. These results should stimulate discussion and research on whether individuals after previous SARS‐CoV‐2 infection would benefit from a two‐part vaccination schedule or whether these currently much‐needed second doses could be saved.

## 1 INTRODUCTION

The SARS‐CoV‐2 vaccine shortage is challenging policymakers and their experts in many regions of the world. This inevitably resulted in the question of who should be vaccinated first.^1^ Although there are no differentiated vaccination recommendations for individuals with previous SARS‐CoV‐2 infection and those who are still naïve to SARS‐CoV‐2,^2^ the question arises from an immunological point of view whether the antibody formation of these two groups differs.

There is indeed evidence that seropositives might exhibit a more pronounced antibody response after the first dose of the vaccine.^3^ In two recent pre‐print articles, it was reported that individuals with both previous asymptomatic and symptomatic SARS‐CoV‐2 infections showed a more pronounced antibody response than naïve individuals did.^4^, ^5^ However, there are so far no data using CE‐marked, automated platform assays for quantification of SARS‐CoV‐2 antibody responses.

Most quantitative SARS‐CoV‐2 antibody assays detect antibodies against the viral spike protein (S) or parts thereof. In pre‐vaccination times, these assays were developed because anti‐S antibodies were shown to correlate well with the presence of neutralizing antibodies.^6^ Currently available vaccinations are based on the induction of anti‐spike protein antibodies by introducing mRNA (e.g., Pfizer and Moderna) or DNA (e.g., AstraZeneca, Johnson & Johnson and CanSino Biologics) coding for it.^7^ Therefore, the presence of anti‐S antibodies might indicate either a vaccination response or a previous infection. However, a further distinction can be made, since a natural infection with the SARS‐CoV‐2 virus induces antibodies against other viral compounds, for example the viral nucleocapsid (NC), which can be detected by CE‐marked immunoassays as well.^8^ Therefore, even after vaccination with one of the mentioned vaccines, it is possible to distinguish those with a previous SARS‐CoV‐2 infection from individuals who were seronegative before their first shot.

The present study aimed to identify seropositives among 81 individuals who received a single dose of BNT162b2 and to compare their antibody response with naïve individuals.

## 2 METHODS

### 2.1 Study design

In this observational study, we compared antibody responses to the first dose of BNT162b2/Pfizer/BioNTech vaccine in individuals previously infected with SARS‐CoV‐2 and SARS‐CoV‐2–infected naïve individuals. Therefore, we included sera of 81 individuals taken 21(±1) days after their first dose of BNT162b2 vaccine. Donors were recruited by the Department of Laboratory Medicine, Medical University of Vienna, in the framework of the healthy donor cohort of the MedUni Vienna Biobank. Inclusion criteria were an age ≥18 years and willingness to give written informed consent, whereas insufficient biomaterial would have led to exclusion from the study. All donors provided written informed consent (Ethics Committee No. 404/2012). The study protocol was reviewed and approved by the ethics committee of the Medical University of Vienna (No. 1066/2021). Reporting of the study conforms to broad EQUATOR guidelines.^9^

### 2.2 Sample management and analytics

Blood samples were centrifuged at 1.884 *g* for 10 minutes at room temperature, and sera were stored for <7 days at 2‐10°C or at <−70°C (if not used immediately) in the MedUni Wien Biobank, a central facility with certified quality management (ISO 9001:2015).^10^

The following tests quantifying antibodies against viral spike protein (S) were used:

(i) the Roche SARS‐CoV‐2 S total antibody electrochemiluminescence sandwich assay (ECLIA) using RBD (receptor‐binding domain) as the antigen on cobas^®^ e801 analyzer series (Roche).^11^ The quantification range is between 0.4 and 250.0 BAU/mL (binding antibody units, referenced to the International WHO Standard NIBSC 20/136). Samples exceeding the upper limit of quantification (ULQ) were analysed in on‐board 1:10 or manual 1:100 dilutions. If results were still above ULQ, they were fixed at 25 001 BAU/mL. The manufacturer states intra‐ and interassay precision between 1% and 3%, a clinical specificity of 99.98% (99.91‐100) and a cumulative sensitivity ≥14 days after the first positive PCR of 98.8% (98.1‐99.3) if 0.8 BAU/mL is used as a cut‐off. (ii) The DiaSorin SARS‐CoV‐2 S1/S2 IgG chemiluminescence assay using S1 and S2 domain as antigens on a LIAISON (DiaSorin).^12^ The quantification range is between 3.8 and 400.0 AU/mL. Samples exceeding the upper limit of quantification (ULQ) were analysed in on‐board 1:10 dilutions. Intra‐ and interassay precision is below 4%, and according to the manufacturer, specificity among blood donors is 98.5% (97.5‐99.2) and sensitivity is 97.4% (86.8%‐99.5%) >15 days after diagnosis at a cut‐off of >15 AU/mL, whereby results between 12.0 and 15.0 AU/mL are considered borderline. (iii) The GenScript surrogate virus neutralization test (sVNT) is based on the principle of measuring the capacity of a patients’ serum to inhibit RBD binding to immobilized ACE receptors. A FilterMax F5 Multi‐Mode Microplate Reader (Molecular Devices) was used for plate measurements. The manufacturer's instructions cites 30% inhibition as an appropriate threshold for positivity; however, a recent publication suggests 20% inhibition as the optimal cut‐off value.^13^

SARS‐CoV‐2 serostatus (seronegative or seropositive) was determined using the Roche anti‐SARS‐CoV‐2 ECLIA, which detects antibodies to nucleocapsid (NC) antigen with 1.000 COI as the cut‐off for positivity. These antibodies are not induced by vaccination with BNT162b2 and therefore highly specific (99.7%) for a past infection with SARS‐CoV‐2.^8^

### 2.3 Statistical analysis

Data are presented as median and interquartile range, unless otherwise indicated. Group differences are assessed by the Mann‐Whitney *U* tests or, to control for age, by ANCOVA. Correlations are calculated according to the Spearman test. The effect of age on group differences was evaluated by analyses of covariance (ANCOVA). Main effects of serostatus before vaccination and number of received doses (1 vs 2), as well as a possible interaction between those variables, were assessed by general linear models with repeated‐measures design. All calculations were performed using the MedCalc 19.2 (MedCalc bvba) or SPSS 26 (IBM), and graphs were drawn with the Prism 9.0 (GraphPad).

## 3 RESULTS

### 3.1 Increased antibody levels in seropositives after the first shot

Of the 81 vaccinated individuals, 11 yielded positive results in the Roche NC ECLIA, indicating a previous infection with SARS‐CoV‐2. A further individual had a negative NC ECLIA (0.220 COI); however, the value was above the reduced threshold associated with the ECLIA Youden index (i.e., >0.165 COI)^14^ and the donor had a PCR‐proven SARS‐CoV‐2 infection in 2020. 69 individuals yielded Roche NC results between 0.059 and 0.106 COI and were therefore considered naïve.

Among the 12 seropositives, Roche NC ECLIA values ranged from 0.220 to 91.800 COI, with a median of 21.630 COI [5.845‐31.100]. Seropositives did not significantly differ in terms of age (seropositives: 42 years [27‐49] and naïve individuals: 43 years (31‐52], *P* = .366) or sex (seropositives: n = 30 females [43%] and naïve individuals: n = 5 females [42%], *P* = .908).

**FIGURE 1:**
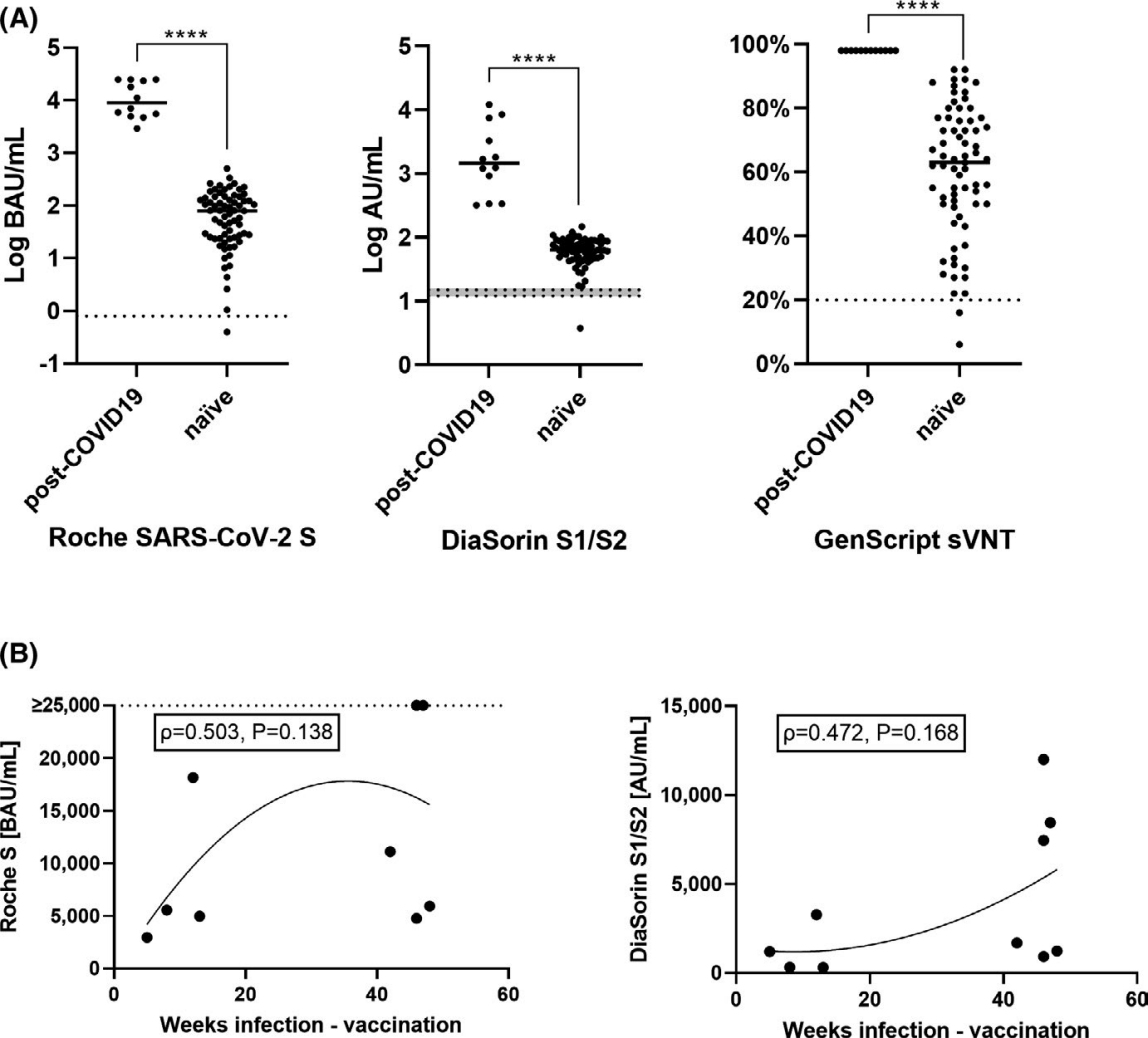
A, Comparison of antibodies against SARS‐CoV‐2 spike protein (S) components measured with three different assays (Roche: receptor‐binding domain (RBD) ECLIA; DiaSorin: S1/S2 combination antigen CLIA; and GenScript: surrogate viral neutralization test (sVNT) with RBD as antigen), in response to vaccination with BNT162b2 in 5 individuals with previous SARS‐CoV‐2 infection (‘post‐COVID‐19’) and 69 individuals without evidence of previous SARS‐CoV‐2 infection (‘naïve’). Horizontal solid lines represent medians. Dotted lines represent cut‐offs for positivity (grey area: borderline results). B, Correlation between the time from infection to vaccination and antibody levels among seropositives. The dotted line marks samples with results that were not further diluted (>25 000 BAU/mL). ****P < .0001

When comparing anti‐S antibodies between seropositive and naïve individuals, the circulating antibody levels were markedly elevated in those with a previous SARS‐CoV‐2 infection (Roche S: 9078.5 BAU/mL [5267.0‐24 298.5] vs 79.6 [24.7‐142.3]; DiaSorin S1/S2: 1465.0 AU/mL [631.0‐5365.0] vs 63.7 [47.8‐87.5]; and sVNT: 98% [98‐98] vs 63 [49.8‐76.3], all *P* < .0001; see Figure 1A).

In 10 of 12 seropositive individuals, the SARS‐CoV‐2 infection was documented, with the earliest detected infection registered in 03/2020. There was a trend for a correlation between the time from infection to vaccination (42 weeks [11‐46]) and the antibody response, for both the Roche S (*ρ* = 0.503, *P* = .138) and the DiaSorin S1/S2 assay (*ρ* = 0.472, *P* = .168), showing higher antibody responses for infections that occurred longer ago, although this relationship was not statistically significant. However, the amount of the Roche NC assay results (n = 12) did not correlate with the Roche S (*ρ* = 0.321, *P* =.309) or the DiaSorin S1/S2 (ρ = 0.109, *P* = .737) response (see Figure 1B).

### 3.2 Antibody response in naïve individuals is associated with age

Age tended to be negatively associated with antibody responses, meaning that older individuals presented with lower antibody levels three weeks after SARS‐CoV‐2 vaccination in initially naïve individuals (Roche S: *ρ* = −0.23, *P* = .055; DiaSorin: *ρ* = −0.28, *P* = .020; and sVNT: *ρ* = −0.22, *P* = .072).

**FIGURE 2:**
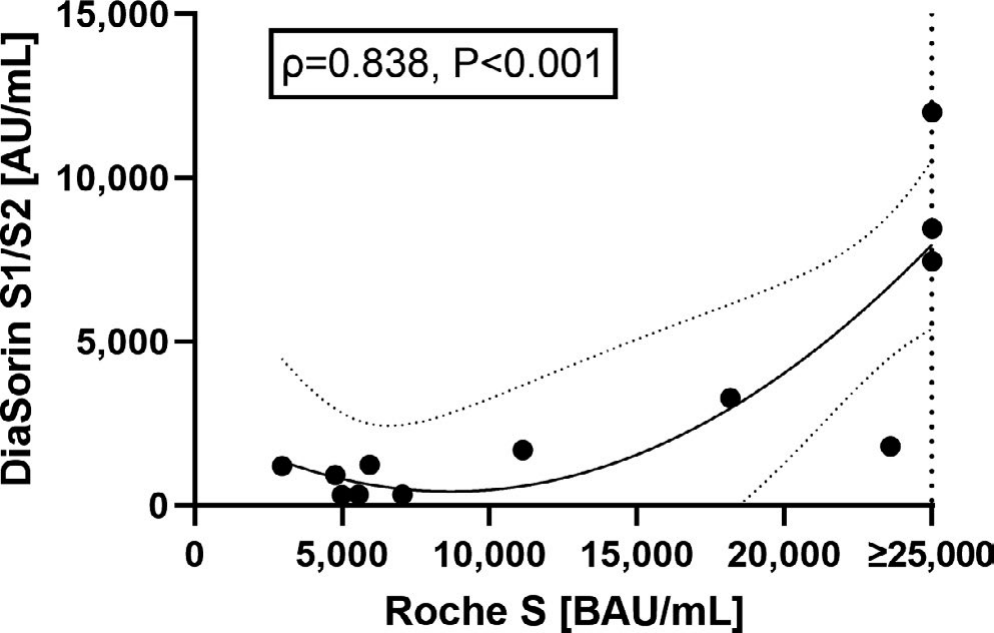
Estimated marginal means (EMMs) and their standard errors (SEM) of SARS‐CoV‐2 antibody responses in SARS‐CoV‐2 seropositives and naïve individuals. For calculation of EMMs, age was kept constant at 42.8 years

To rule out a possible effect of age on the association between SARS‐CoV‐2 serostatus and antibody levels after the first vaccination, we performed ANCOVAs. However, the stronger antibody response of individuals following SARS‐COV‐2 infection observed in univariate analysis could not be vanished by including age as a covariate. The mean difference between seropositives and naïve individuals was 13 237 BAU/mL (95% confidence interval, CI: 11 058‐15 417) for Roche S, 3260 AU/mL (95% CI: 2360‐4159) for DiaSorin S1/S2 and 36% (95% CI 25‐48) for sVNT (Figure 2). Indeed, age was only a significant predictor in the ANCOVA assessing sVNT values (F = 4.88, *df*_1_ = 1, *df*_2_ = 78, *P* = .030) and showed no significant moderating effect on the association between Roche S or DiaSorin S1/S2 and SARS‐CoV‐2 serostatus.

**FIGURE 3:**
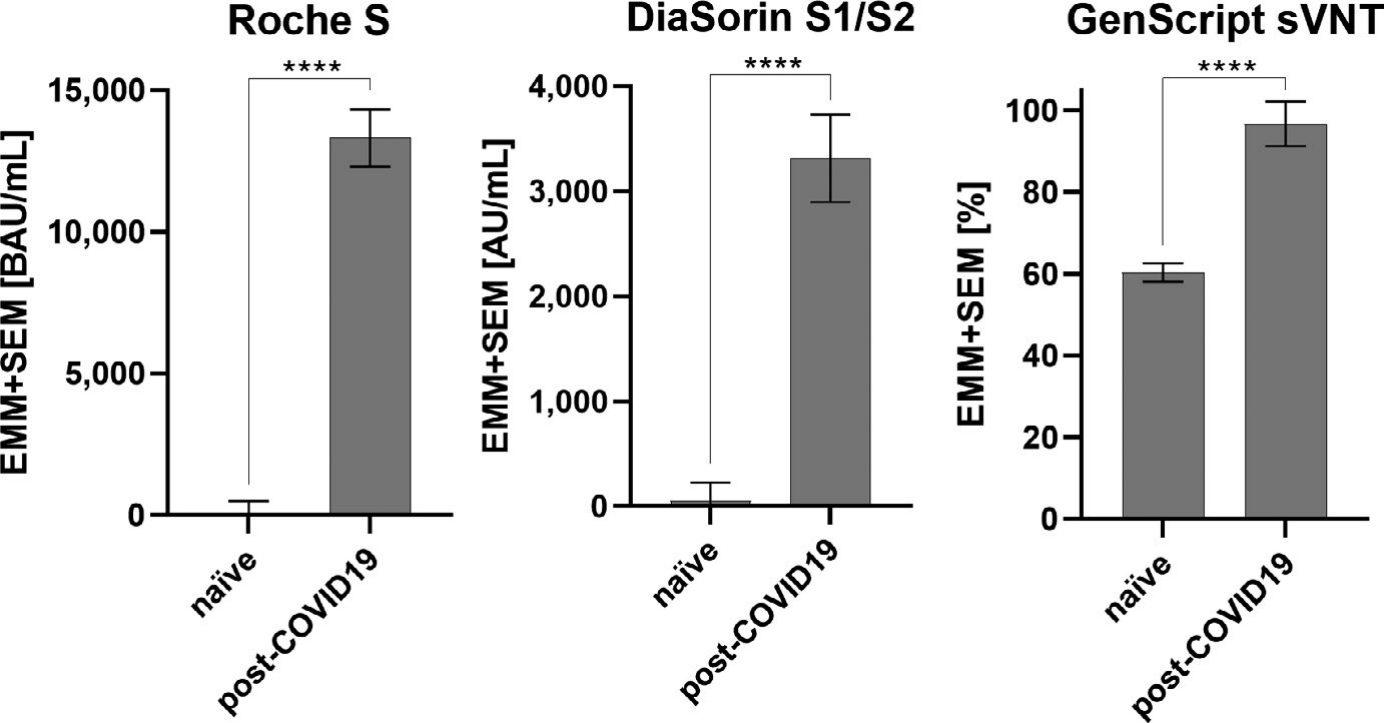
Correlation between anti‐spike protein antibodies quantified by the Roche S or the DiaSorin S1/S2 assay among seropositives (n = 12). The dotted vertical line marks samples with results that were not further diluted (>25 000 BAU/mL)

### 3.3 Comparing the antibody levels of the two different antibody binding tests in SARS‐CoV‐2 seropositives

Anti‐S antibody levels measured by the Roche S and the DiaSorin S1/S2 assays were compared among seropositives in order to evaluate the reliability of our findings. The results from both test systems were in good overall agreement (ρ = 0.838, *P* < .001; Figure 3), suggesting that the variability observed represent genuine differences in antibody reactivity.

### 3.4 Effect of the second dose on antibody levels

**FIGURE 4:**
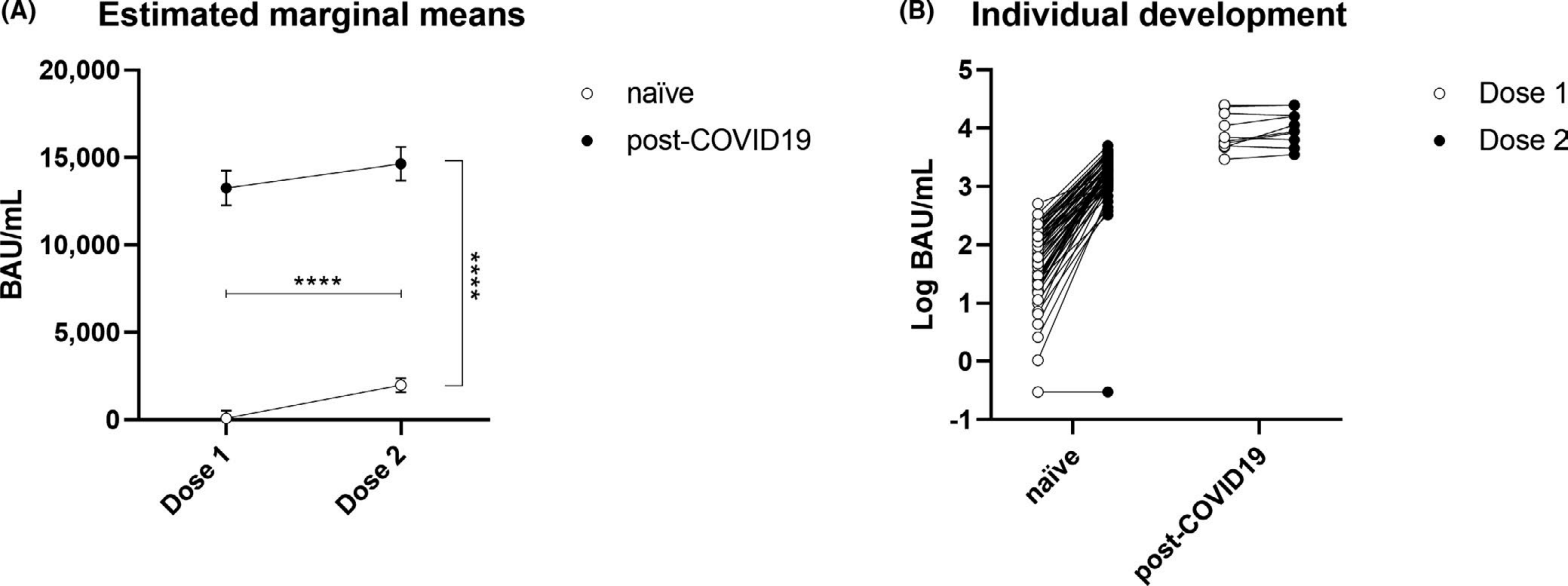
A, Estimated marginal means ± standard errors of antibody levels (Roche SARS‐CoV‐2 S) after BNT162b2 doses 1 and 2 in both naïve (open circles) and SARS‐CoV‐2 seropositive (filled circles) individuals, revealing significant main effects for dose and seropositivity (****P < .0001). B, Individual developments of SARS‐CoV‐2 antibody levels (Roche SARS‐CoV‐2 S) after BNT162b2 doses 1 (open circles) and 2 (filled circles) in both naïve and SARS‐CoV‐2 seropositive individuals

For 68 of the 69 SARS‐CoV‐2‐infected naïve donors and all 12 post‐COVID subjects, blood samples collected 3‐6 weeks after the second dose of BNT162b2 were analysed by the Roche SARS‐CoV‐2 S assay. A general linear model with repeated‐measures design was used and revealed significant main effects for the second dose and pre‐vaccination infection status: F = 60.8, *P* < .0001; F = 149.8, *P* <.0001. Specifically, SARS‐CoV‐2‐infected naïve individuals increased from 79.6 [24.7‐142.3] BAU/mL to 1840.0 [1143.5‐2666.5 BAU/mL, and antibody levels of those with prior infection increased from 9078.5 [5267.0‐24 298.5] BAU/mL to 13 809.5 [7344.0‐>25 000] BAU/mL after the second dose (see Figure 4). Although this increase did not differ between groups in absolute terms, the relative changes in antibody after the first dose increased by 25‐fold [15‐56] compared with those after the second dose in the naïve individuals, while the antibody levels of those previously infected remained more or less the same (1.0 [1.0‐1.5], difference = *P* < .0001).

## 4 DISCUSSION

Our data clearly show that individuals with previous SARS‐CoV‐2 infection, as indicated by circulating antibodies against the viral nucleocapsid, respond considerably better after a single dose of BNT162b2 than naïve individuals did. We report this here for the first time using two different commercial CE‐labelled automated electrochemiluminescence assays.

Astonishingly, depending on the binding assay used, a more than 100‐fold (Roche) or 20‐fold (DiaSorin S1/2) increase in median antibody levels of seropositive versus seronegative individuals could be shown. In particular, the Roche assay showed excellent discriminatory power between these two groups. The highest measured antibody level of SARS‐CoV‐2‐infected naïve individuals is 508 BAU/mL, whereas the lowest of seropositive individuals is 2948 BAU/mL. Furthermore, all 12 seropositive individuals showed the maximal inhibition of 98% of ACE receptor binding in the functional sVNT assay. Although this functional neutralization surrogate assay clearly shows differences between subjects without and with prior SARS‐CoV‐2 infection in the present cohort, this is no longer expected after the second dose of vaccination. Thus, this assay is more suitable for principal detection of functional neutralizing antibodies after vaccination than for reliable quantification and discrimination of high antibody levels.

Our observations are consistent with those reported in a recent correspondence published in The Lancet by Prendecki et al^15^, who showed that individuals with suspected natural infection yielded higher anti‐S levels 21 days after the first dose, as measured by the Abbott II IgG assay. The differences in anti‐S levels between naïve and seropositive individuals were highly significant, but there were overlaps. These overlaps might be explained by the following: (i) not only individuals positive in the Abbott NC assay but also seronegatives with sole T‐cell responses to nonspike antigens were included. (ii) The chosen NC immunoassay from Abbott is known to be highly prone to antibody waning, and those who were infected in early 2020 could be falsely classified as seronegative.^16^, ^17^, ^18^ Random testing of our positive cohort with the Abbott NC test showed that only about half of the Roche NC seropositives were also positive in the Abbott NC test (data not shown), although all Roche NC seropositives presented with remarkably higher antibody responses after vaccination than naïve individuals. Furthermore, in the cohort studied here, people who had been infected earlier tended to respond better to vaccination.

Our findings are also in line with data reported as pre‐print articles,^4^, ^5^, ^19^, ^20^ showing that anti‐S IgG was higher among individuals with previous SARS‐CoV‐2 infection than in those without. However, the reported individuals received either BNT162b2 (Pfizer/BioNTech) or mRNA‐1273 (Moderna), no CE‐labelled in vitro diagnostics were used for antibody quantification, and in three of the articles, serostatus was assessed from patient records and not verified by measuring natural infection‐specific antibodies, bearing the possibility of including false positives or false negatives.

A further indicator for the protective effect of a natural SARS‐CoV‐2 infection is the low observed rate of reinfections.^17^, ^21^, ^22^ Individuals who underwent SARS‐CoV‐2 infection are thus generally considered immune for at least a few months. It could therefore, be hypothesized that a single bout of a SARS‐CoV‐2 vaccine might be sufficient for consolidating this protective immunity. As indicated in recent pre‐print articles, seropositives might reach very high antibody levels already one or two weeks after the first dose.^4^, ^5^ Since most available vaccines induce only antibodies against the viral S protein, it might not be mandatory to assess the serostatus in advance; testing for both anti‐NC and anti‐S antibodies, approximately 14 days after the first shot, could assist physicians in deciding whether a second dose is necessary or could be postponed or even omitted. The suggested algorithm is further supported by the observed changes in antibody levels after the second dose of vaccination. Although both groups showed increases in antibody levels after the second dose, the seropositive subjects did not show relevant relative changes and still were on average significantly above the SARS‐CoV‐2‐infected naïve participants. SARS‐CoV‐2‐infected naïve individuals, on the other hand, showed a multiplication of their antibody levels after the second dose, but still could hardly reach the levels of previously infected participants.

This manuscript comes with several strengths and limitations. Strengths are the use of CE‐labelled, well‐documented antibody assays, which increases the reliability of the data, and the similar distribution of both seropositive and naïve individuals in terms of age and sex. The current limited number of seropositives in this study could be seen as a limitation.

In conclusion, we here showed that the antibody response in seropositives after the first BNT162b2 dose is markedly higher than that in individuals without a previous SARS‐CoV‐2 infection, using CE‐labelled in vitro diagnostic tests. With these data, we hope to stimulate discussion and research on whether individuals after previous SARS‐CoV‐2 infection or known seropositivity would benefit from a two‐part vaccination schedule or whether these currently much‐needed second doses could be saved.

## CONFLICTS OF INTEREST

NP received a travel grant from DiaSorin. The Dept. of Laboratory Medicine received compensations for advertisement on scientific symposia from Roche, DiaSorin and Abbott, and holds a grant for evaluating an in vitro diagnostic device from Roche. The GenScript sVNT Kit was kindly provided by the supplier (medac GmbH). MedUni Wien Biobank is part of the Austrian biobank consortium BBMRI.at. There was no additional funding received for the present work.
